# Supplementary figures and images for: Cbf11 and Mga2 function together to activate transcription of lipid metabolism genes and promote mitotic fidelity in fission yeast
Source: PLoS Genet. 2024 Dec 9;20(12):e1011509. doi: 10.1371/journal.pgen.1011509 (PMC11658701; doi:10.1371/journal.pgen.1011509)

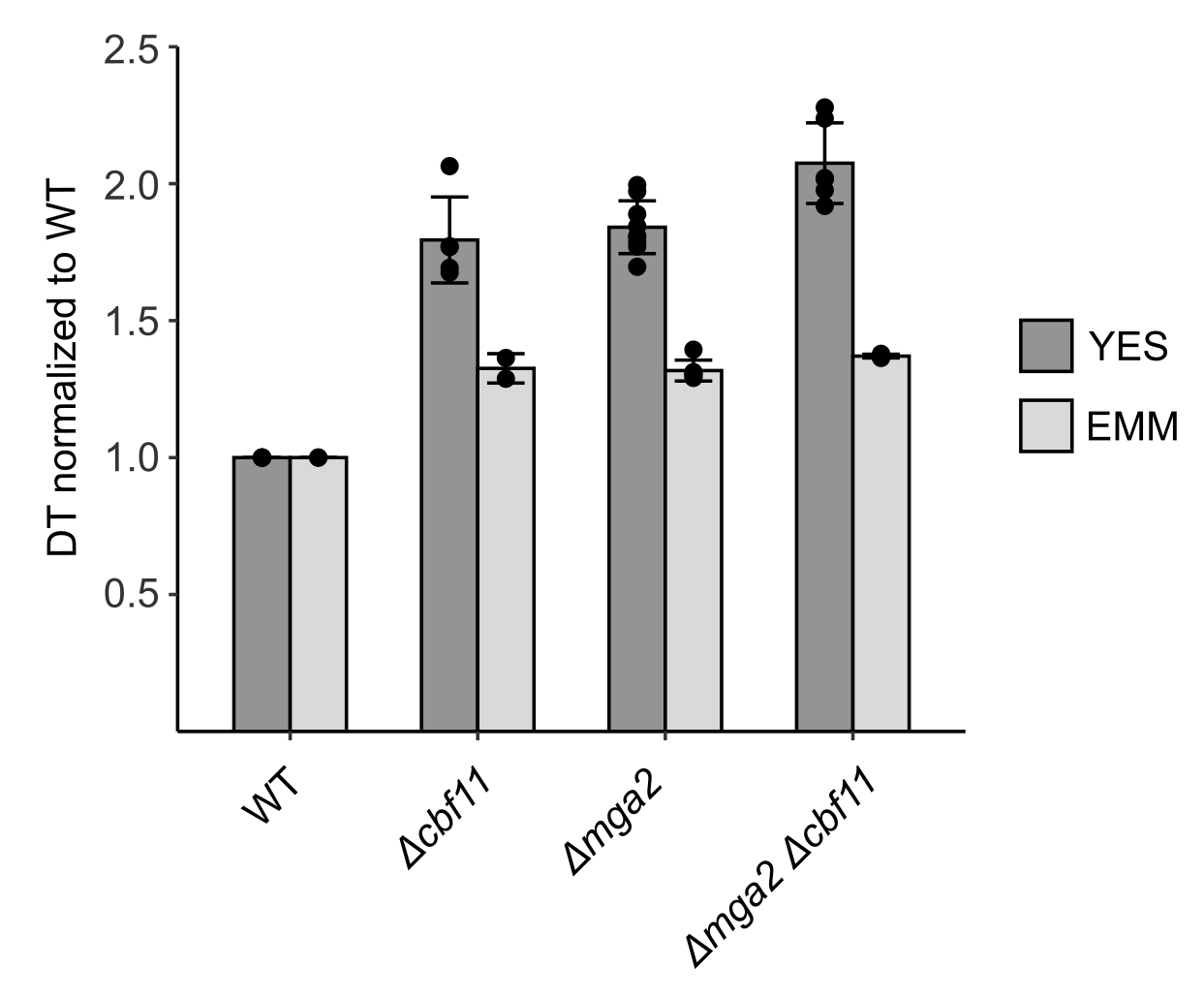

Supplement: S1 Fig — These defects are partially suppressed in the EMM minimal medium. Mean ± SD values, as well as individual data points for ≥2 independent experiments are shown. (TIFF) [file pgen.1011509.s001.tiff]

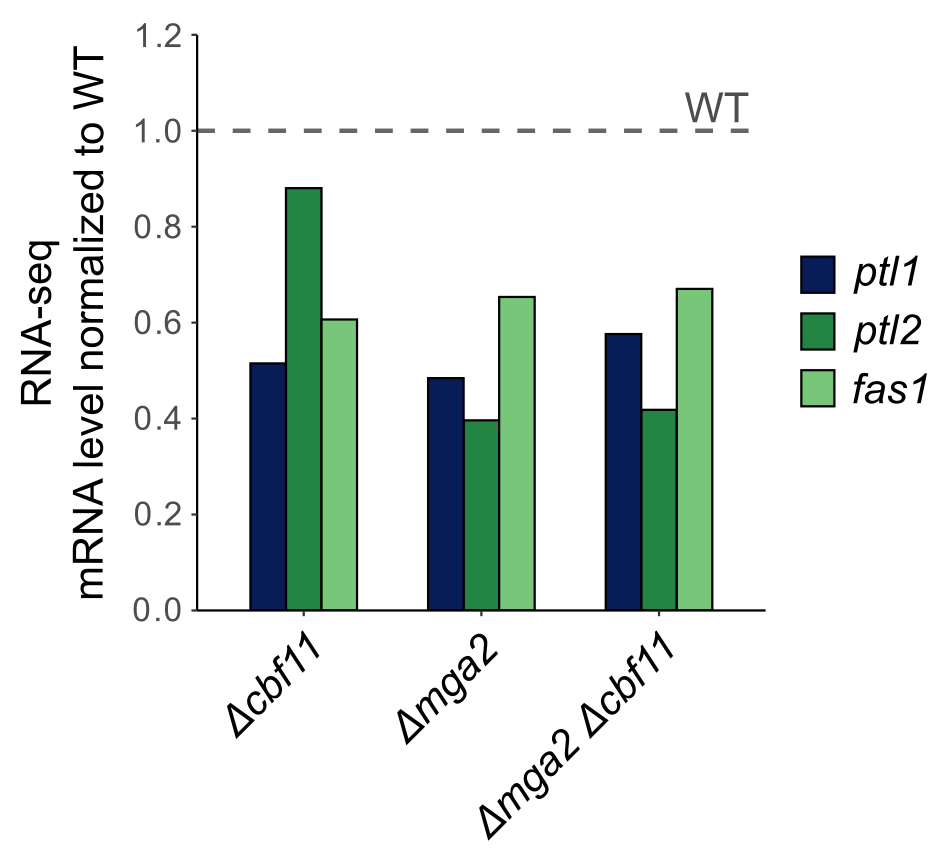

Supplement: S2 Fig — The ptl1 and ptl2 genes encode triacylglycerol lipases, the fas1 gene encodes the fatty acid synthase alpha subunit. Mean values for 3 independent experiments are shown. (TIFF) [file pgen.1011509.s002.tiff]

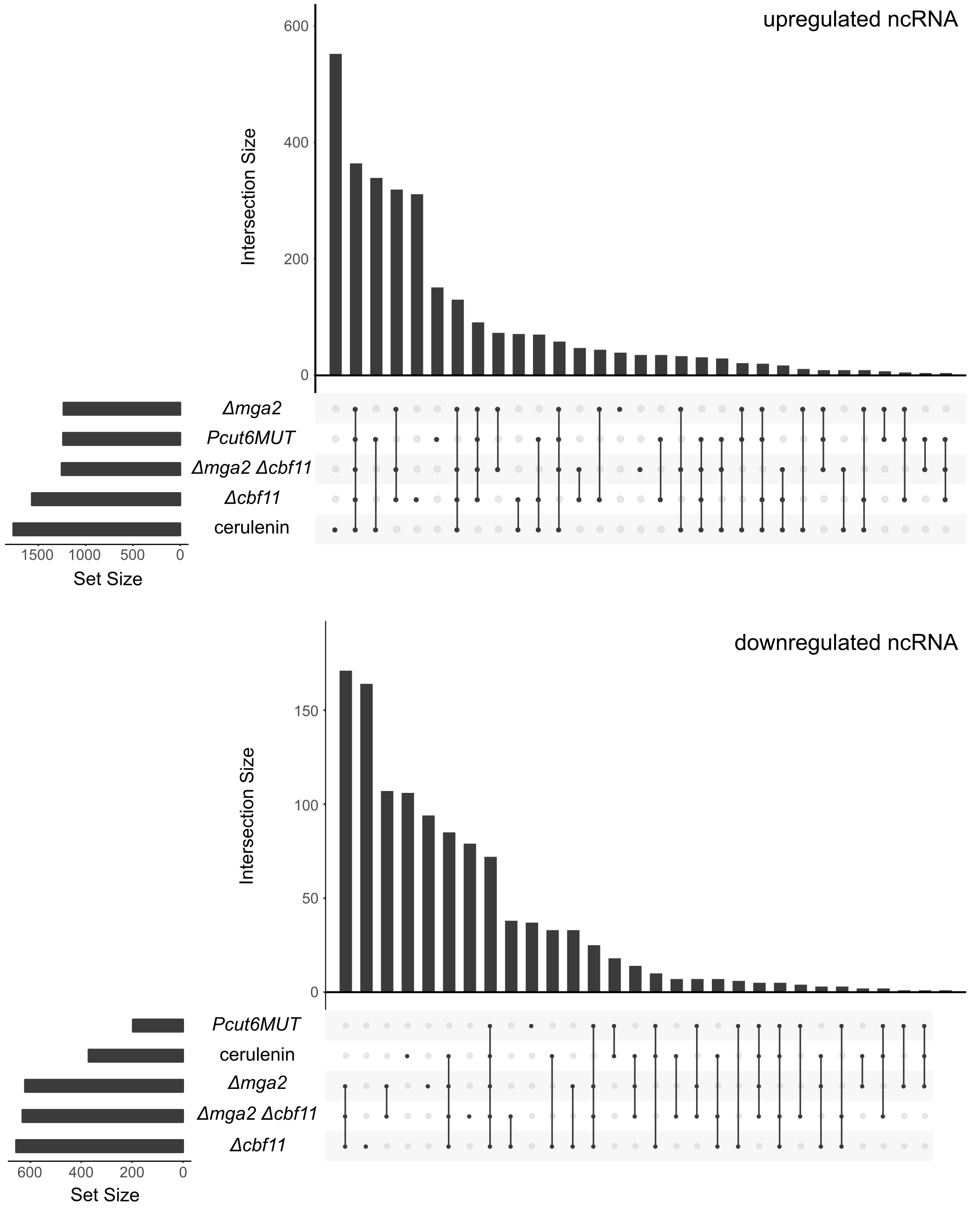

Supplement: S3 Fig — Sets of ncRNAs upregulated (top panel) or downregulated (bottom panel) in the Δcbf11, Δmga2, Δmga2 Δcbf11 or Pcut6MUT mutants, or upon cerulenin treatment were subdivided based on their mutual overlaps. The sizes of the intersections were plotted, with subset membership indicated below the plot. The results show that hundreds of ncRNAs are jointly upregulated under all conditions tested. The figure was created using the UpSetR package in R [59]. (TIFF) [file pgen.1011509.s003.tiff]

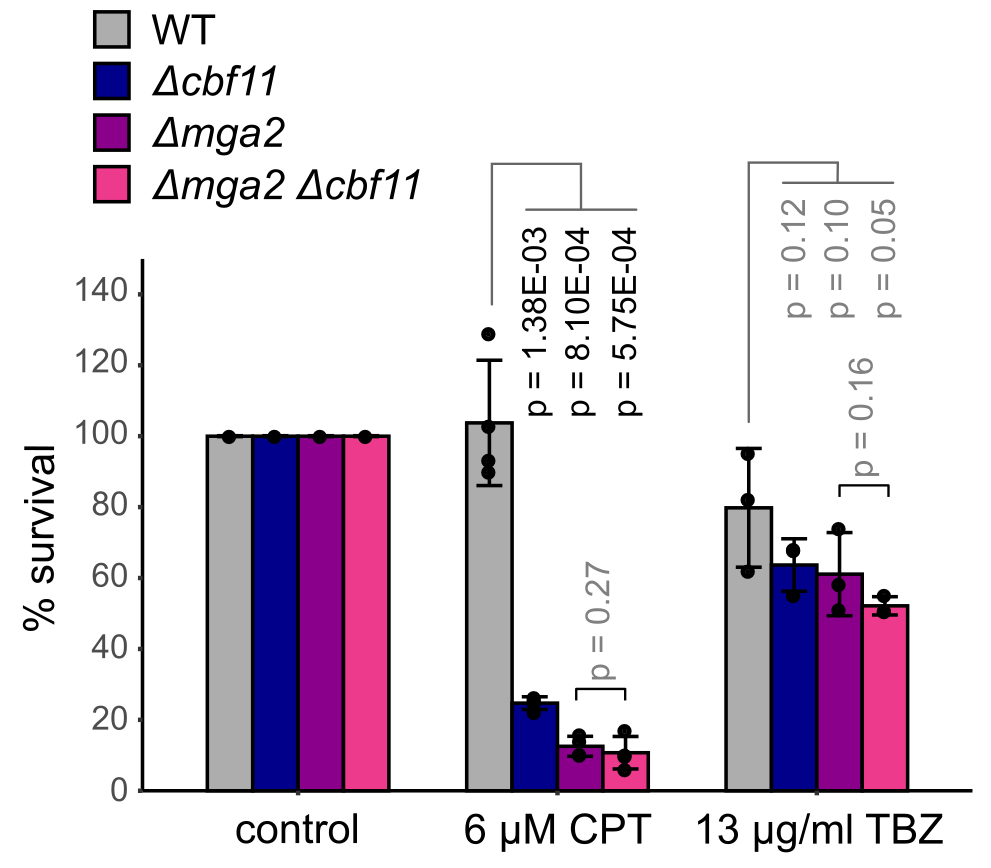

Supplement: S4 Fig — Quantification of colony-forming units upon chronic exposure to indicated concentrations of CPT and TBZ is shown as % survival. Mean ± SD values, as well as individual data points for 3–4 independent experiments are shown. Significance was determined by an unpaired one-sided Welch Two Sample t-test. (TIFF) [file pgen.1011509.s004.tiff]

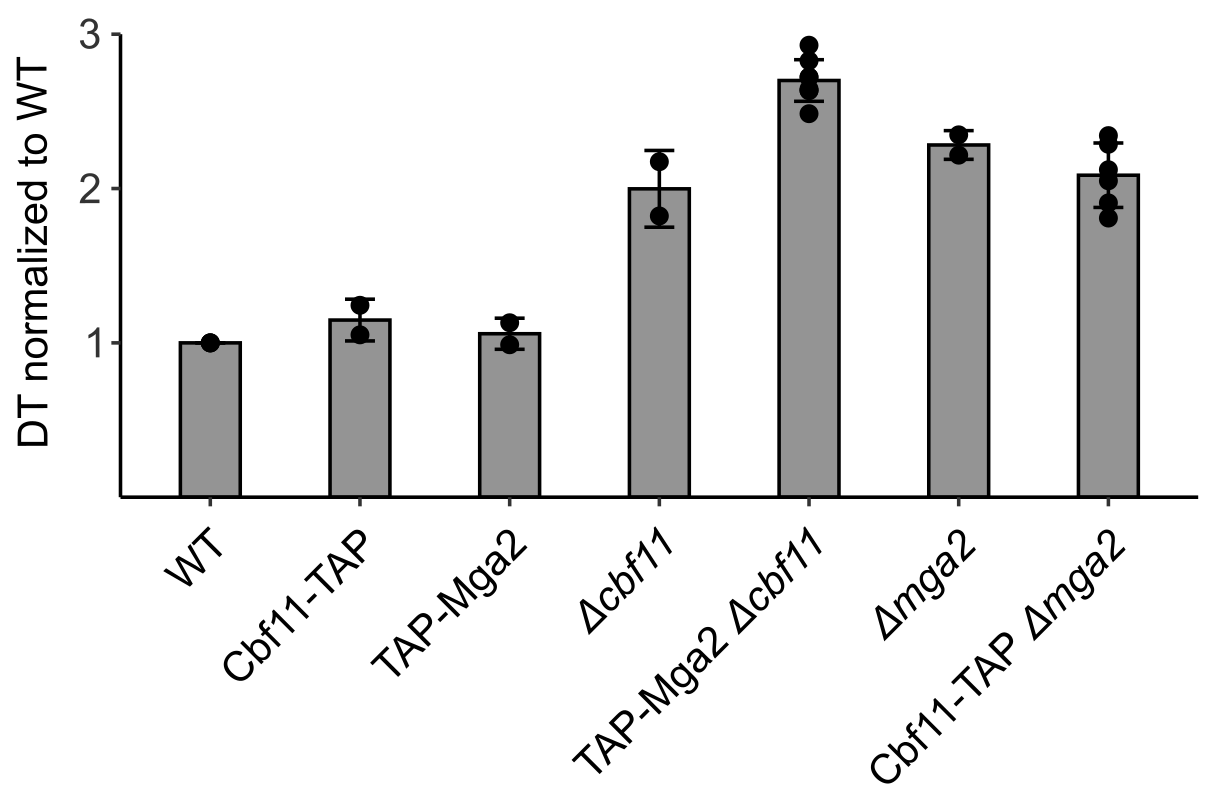

Supplement: S5 Fig — Mean ± SD values, as well as individual data points for ≥2 independent experiments are shown. (TIFF) [file pgen.1011509.s005.tiff]

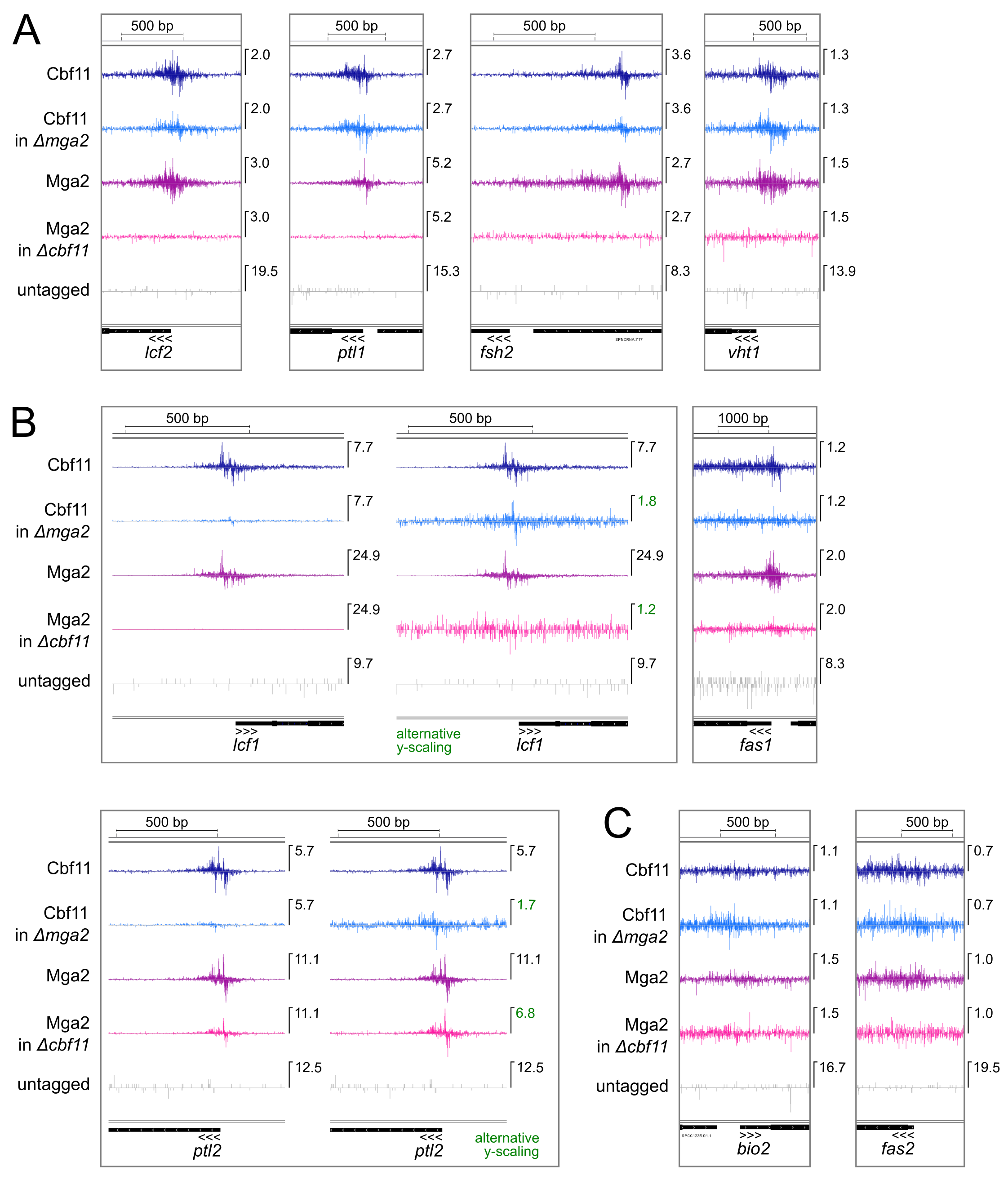

Supplement: S6 Fig — (A) Promoters to which both Cbf11 and Mga2 bind, and the binding is Cbf11-dependent. (B) Promoters where binding shows varying dependence on Cbf11 and/or Mga2. (C) Control loci with no binding. Mean strand-specific coverage profile of 3 independent experiments for Cbf11 and Mga2 in the indicated genetic backgrounds, and a strand-specific coverage profile for untagged WT cells (negative control) are visualized in the Integrated Genome Viewer (IGV; Broad Institute). Gene orientation is indicated by three arrowheads; the maximum Y-axis value for each track is indicated on the right side of each panel. (TIFF) [file pgen.1011509.s006.tiff]
