# Supplementary material for: Cbf11 and Mga2 function together to activate transcription of lipid metabolism genes and promote mitotic fidelity in fission yeast
Source: PLoS Genet. 2024 Dec 9;20(12):e1011509. doi: 10.1371/journal.pgen.1011509 (PMC11658701; doi:10.1371/journal.pgen.1011509)
Supplement: S1 Text — (DOCX) [file pgen.1011509.s007.docx]

**S1 Text: *TAP-mga2** template for HR**

*SacI* - *mga2* 5’ upstream - *mga2* 5’UTR - *NotI* - TAP (w/o STOP codon) - *XbaI* - linker - *PstI* - *mga2* ORF with silent point mutation * (C→A) - *XhoI*

GAGCTCGGGGGTCCAAGAGCAAATGTTGCAAGTGCTGCGAAATTTTACATGTCAGAAAGAAGAAAGCGTGGATTTTCTTTTGAAAATGGTTCCTATGGAGTTGTTAGCAAAAATTTTGCTTGAAAAATTAGAAAGTAAAAATCCTATCCTAATTGAACCCAGTATTTACATTTTGGTGCACATCGCTGCCAGCGATGGTGAGCTTAGGGATTCTATCCTAAGGCAAACCAAACTACTACTATTGGTGAAGGAAATTATGCTACAAGAAGCACAACGTCTCAAGCCCGAGCAAATGTCCACAAATGTGTCATCTCCGGAAAGTGACAGTGAAATGGATTTGCAAGAAGATGATTTTGATAGGGAAATGCGTCCTCCTTTCGATATTTATGAAGAGGACACTTATGAACCAAACTCCGAAATTCTGTTGGCCGGAATTTGGCTCTGTATTAACATTCTATGGCCCAAACAATGTACATCTCCAAGCCAGGAAGATAAGGAAAGAGCAAGTATCTTACAAAATCTCGGATTTGGAGAATGTTTGCAGATGCTACAAAATCACTCATCTCCTGATGTAAGGGAACGTGTCAAAGATGCCTTGATGTACATCAATGTAACCGATTAATTTCTATTACGCTTTTCCGTTGTCTTTGCCGACTTGATTGGAACAGTCTAACAAATCGGAGCCATTTTCGCATTTAGTATTTCTGTATGTATAAATGTGTGCACAACTCTTGCAGCTGAAGTTAACAGGTTGTTGGATTGAAGTACTTTGAGAATGATCGTATATAAAAAGATTTAATGATTTATCAAAACGAATAAAATAAGTATTATGAACATTCAACTTATATGTATGCGGGATAGTGTAAAAACCTATTTAACATTGTAAAAATTTTTTAAGGAAAGTTGTACTTTGACAAGAACAATTTCATTTACATGTATTCTACTAAATTTATCAGAATGTTGGGAATTTTTTTGTTGGTATATAATGCCGAAAGGAAATTAATCGAATATGAAGAAAGTGGCGGAACTAAAAAACAAAACCCACAAACTAATTATTTAAATAAATATAACATAATAACTTGTTAAAATAAGAGTAATACTAGAGAATGTGTGCTCAAGTGGAAAGCATGTAATACTTGCACAACGTGATGGAAGAGATTAGTGTAGTAGCGCAGCGACAGCTTCCAACAACCAACTAATCGAGGAGGAAAGCAAAAGTGAGAATTGAAAAGAAGTAGTACATTAAAGACCTTTAAATTCTTTGAAAGAATTGCCTTTATAAAGGTAGTCTATTGCATTTCAAAGTACTCTCAAGGCGGCCGCATGAAGCGACGATGGAAAAAGAATTTCATAGCCGTCTCAGCAGCCAACCGCTTTAAGAAAATCTCATCCTCCGGGGCACTTGATTATGGTACCCCAACTACTGCTTCTGAAAATCTATATTTTCAAGGTGAACTAAAAACTGCTGCTTTGGCTCAACATGCGATTAAGGGTGAAGCTCAAAAACTTAATGACTCTCAAGCTCCAAAAGCTGATGCGCAACAAAATAACTTCAACAAAGATCAACAAAGCGCCTTCTATGAAATCTTGAACATGCCTAACTTAAACGAAGCGCAACGTAACGGCTTCATTCAAAGTCTTAAAGACGACCCAAGCCAAAGCACTAACGTTTTAGGTGAAGCTAAAAAATTAAACGAATCTCAAGCACCGAAAGCTGATAACAATTTCAACAAAGAACAACAAAATGCTTTCTATGAAATCTTGAATATGCCTAACTTAAACGAAGAACAACGCAATGGTTTCATCCAAAGCTTAAAAGATGACCCAAGCCAAAGTGCTAACCTATTGTCAGAAGCTAAAAAGTTAAATGAATCTCAAGCACCGAAAGCGGATAACAAATTCAACAAAGAACAACAAAATGCTTTCTATGAAATCTTACATTTACCTAACTTAAACGAAGAACAACGCAATGGTTTCATCCAAAGCCTAAAAGATGACCCAAGCCAAAGCGCTAACCTTTTAGCAGAAGCTAAAAAGCTAAATGATGCTCAAGCACCAAAAGCTGACAACAAATTCAACAAAGAACAACAAAATGCTTTCTATGAAATTTTACATTTACCTAACTTAACTGAAGAACAACGTAACGGCTTCATCCAAAGCCTTAAAGACGATCCTTCGGTGAGCAAAGAATCTAGAGGTTCTGGTGGTTCTGGTCTGCAGATGAATTTAGAATTTTCTTTTGGAAATTTGATCGCCAAAAATACAGAGAATTCGACTAAGGAAGAAGGAACTTGGGAAGTAAACAAGCTCATGAAATTTAGTTCAGAGGAGGGGAGGTCTTCCTCTGATGATTATATGTTTTCCTCTCCCGATTTTGAAAAAGCCGGAAACGGCGATGCAGAAATGCGTGAATTTTTTAACTTCGATGGATTGCCGGATCAAGGTCTAAATTTGCCTAGTATAGCTCCTCCGTCTTTATCCCATGCCAGTTCACCTAACTTATCGAATTCACAAGATGAAGCGGAATGTTTACCGTCTGATCGTCAGCAAGACTACATCAATCCTAGTTTGCATTTGAACAGAACGGTGTTTCCTACTCCGCAACATTCTATTTCTGATGCTAATTTTCTAGCTAATACCGTTGATCAACCTTTGGGAGATAATCCCATGTTTGGAGAGTCGGATGTTTATCTTTTAAAAATGGATCCTATGAAACAAGCACCTTATGAAGCTGGATTTAATTCTGTAAAGTCATCGGGCGCCATCGAAGATCCATTACAGTTTAGACAGCCGATTACCATGTTGGAAACTCCCTTCAACGAATCTATTAATACTCTTACGCCATATGCCGAAGATTATGCATTTTCCTCTTTAAATACGTCTGCACCGCCTCTTTCTAATAAGGAGTACGCGTTCTCAGTTAATCATTTACCAGCTATCAATGAGCACAAATGGAAATCTCGGGTGGAAACTAACATGCTTTTTGAGTTGCGGATCAAGAGCAACGATAATCAAAGTGTTCCGTTTGAATATCTTCGCTTGCCAAGTTGGGCTCACCGTGAAGACAAAAAGCGTTCTTCGAAACCTCAGCCTTTGCAACCCGATCCGGAGACCGTCATTCATCTTGTACCTACCGTTCTTGCAGGTGACAAAAGCTCAGTTGTTAAGACTTGCTGTACTCGCTGTTTACTTCGAGAAAGGAAGCGCAATGCTCGATCTCAAGCAACAAAGGATGCTTGTATGCCTAACTATACTAAACTGAAGGCTTATGAGCGTAACATGACTGATGCCTCTCCCGAAGAAAAGCAGCAGTTCAGGATCAAGCTCTTAAATCAATTTCCCAAACTTGAAGACATAGATGAAGATAGAATGATAATGGTTTTTACTGGACCTGAGTACGTTCGTTTGCAATTGGATGGAAATGAAAGAGTTGCTCACATCAATGCACGGATTACATGTTACTCGTCTCATCAATCATGCCCTTACTTTCACATCATATGGGACTTATACTCCATGTCACGTTTGGTGGATCGTCTTGTTTTTCCAGAGCCTGTTACCGTTCTTGATGATCATAAAAGTAGAAATCTTACGAAATCAGAAAAGACTGGTAAGAGCAATTCTCAACAAGCTCCTTCAAACCATGTTCTTTCTAAAAGCAATACTGTCCCCAATCTTGTCACTGGTTTCCCTACCCGTTCAGATAATCCACCAAATGAAAAGCGTCGTCGAACATCCTCGAG
