# Supplementary material for: Cbf11 and Mga2 function together to activate transcription of lipid metabolism genes and promote mitotic fidelity in fission yeast
Source: PLoS Genet. 2024 Dec 9;20(12):e1011509. doi: 10.1371/journal.pgen.1011509 (PMC11658701; doi:10.1371/journal.pgen.1011509)
Supplement: Attachment — Submitted filename: 145666_1_rebuttal_3183139_slprpn.pdf [file pgen.1011509.s010.pdf]

Full Revision

Review COMMONS

Manuscript number: RC-2024-02441
Corresponding author(s): Martin, Převorovský

1. General Statements [optional]

We thank all reviewers for evaluating our manuscript and for their useful feedback. Our responses to the reviewers are below in blue. In the main manuscript file, changes and additions are highlighted with color.

2. Point-by-point description of the revisions

REVIEWER #1

Reviewer #1 (Evidence, reproducibility and clarity (Required)):

In this manuscript, the authors examined the relationships between two transcription factors regulating lipid metabolism in S. pombe, Cbf11 and Mga2. By comparing the phenotypes of the deletion mutants lacking either one of them or both, they convincingly showed that Cbf11 and Mga2 act in the same pathway. Furthermore, the authors analyzed their inter-dependence in binding genomic sites and found that Cbf11 is required for the DNA binding of Mga2. These findings provide useful new insights into the functions and mechanisms of these two proteins.

I have only a few minor suggestions for the authors to consider.

1. In Figure 1A, many cells marked with cyan asterisks do not show the typical "cut" phenotype, where a nucleus is bisected by the septum. It may be better to use arrows to point to the septa that are bisecting the nuclei.

Thank you for your suggestion. We have edited the image accordingly. Currently, cyan arrows point to the cells displaying the ‘cut’ phenotype and indicate positions where a nucleus is trapped in a septum and/or cell wall.

2. In Figure 1A, the three types of morphology phenotypes of a mutant are shown in three separate micrographs. I think it may be better to use one micrograph to show all three types of phenotypes.

We are sorry, but we cannot accommodate this request. It is very rare to find enough cells with all the observed phenotypes close together in the field of view. Therefore, we are inclined to present the phenotypes separately, so that we can show enough examples and provide a comprehensive overview of the range of detected phenotypes.

3. The way that the deregulation of non-coding RNAs is shown in Fig. 2E does not indicate whether different mutants and treatments affect non-coding RNAs in a similar manner. It would be helpful to show a more direct comparison, like the way mutants are compared in Figs. 2C and 2D.

For the visualization of intersecting sets, we have decided to use the UpSetR package in R (PMID: 28645171), which enables clear comparison of deregulated non-coding RNAs between different mutants and/or treatments (shown as Supplementary Figure S3).

4. Figure 3A shows a dramatic cell-to-cell variation in LD content in the mutants. Are there correlations between the LD content of mutant cells and other phenotypes of mutant cells? What may explain this heterogeneity in LD content?

We have previously reported heterogeneity in LD content and shown that *Δcbf11* cells with the 'cut' phenotype are enriched for cells lacking detectable lipid droplets. Moreover, 'cut' *Δcbf11* cells have significantly lower numbers of lipid droplets compared to 'non-cut' *Δcbf11* cells, demonstrating association between the 'cut' phenotype and lipid droplet content in the *Δcbf11* mutant (PMID: 29931271). Unfortunately, we still do not know what underlies the heterogeneity of *Δcbf11* cells.

Referee Cross-Commenting

I agree with the comments of Reviewer #3.
I also feel that it may be better to not make the claim that Cbf11 and Mga2 function as a single entity.

We have rephrased the problematic sentences to emphasize that Cbf11 and Mga2 are closely functionally related and most likely work in the same regulatory pathway. Since we were indeed not able to prove the physical interaction between Cbf11 and Mga2, we now avoid the claim that Cbf11 and Mga2 function as a single entity in the revised version of our manuscript.

Reviewer #1 (Significance (Required)):

This study uncovers a collaborative relationship between Cbf11 and Mga2. This represents a major advance in understanding how these two proteins function. This discovery should be of great interest to scientists studying the regulation of lipid metabolism.

REVIEWER #2

Reviewer #2 (Evidence, reproducibility and clarity (Required)):

Cbf11 and Mga2 are transcriptional activators of lipid metabolism genes. This manuscript describes that mechanisms for Cbf11 and Mga2 to regulate lipid metabolism genes in fission yeast. The single deletion of either cbf11 or Mga2 shows similar phenotypes, and the phenotypes of double deletion are not additive, suggesting that these genes functions in the same pathway. Furthermore, Cbf11 and Mga2 share similar binding sites on the promoters of target gene. Cbf11 binds to DNA on its own, but does not activate the gene without Mga2. Mga2 binding requires Cbf11. Therefore, Cbf11 recruits Mga1 to the target promotors, and Mga2 activate transcription of the target genes. Experiments are carefully executed, and manuscript is well written. Conclusions are supported by the data. I have no serious concerns.

Reviewer #2 (Significance (Required)):

Cbf11 and Mga2 are transcriptional activators of lipid metabolism genes. Cbf11 is also known as a cell cycle regulator. The deletion of cbf11 or mga2 gene shows the "cut" phenotype characteristic of mitotic errors. Cbf11 and Mga2 function in the same regulatory pathway linking lipid metabolism and mitotic fidelity. A link between lipid metabolism and mitotic fidelity is a new aspect, which has not been well studied. It has been increasing importance that lipid homeostasis is important for chromosome stability. These findings are of interest for readers in basic biology of lipid metabolism, chromosomes and genome stability.

REVIEWER #3

Reviewer #3 (Evidence, reproducibility and clarity (Required)):

Fission yeast Cbf11 is a protein belonging to the CSL transcription factor family, which involved in Notch receptor signaling for development in metazoans. The authors previously showed that, in fission yeast lacking the Notch pathway, Cbf11 is involved in cell-cycle dependent transcriptional regulation of the cut6 gene encoding an acetyl-CoA carboxylase (doi.org/10.1080/15384101.2016.1235100), dysfunction of which reportedly causes the fetal 'cut' phenotype (doi: 10.1083/jcb.134.4.949). Mga2 was originally identified as a gene required for adaptation to low oxygen, and found to regulate transcription of genes related to lipid metabolism (10.1074/jbc.M116.723650). While Cbf11 and Mga2 are known to be transcriptional regulation of non-sterol lipid metabolism genes, functional relationship between these proteins remains unclear.

In this study, Maresova and colleagues showed that double deletion of cbf11 and mga2 genes did not cause additive defects, indicating that these genes function in a same pathway. Consistently, transcriptome and proteome analyses revealed that target genes of these transcriptional regulators largely overlap, and ChIP analysis clearly showed that Cbf11 and

Review COMMONS

Mga2 share DNA binding sites and that DNA binding of Mga2 is dependent on Cbf11. Based on these findings, the authors concluded that Cbf11 and Mag2 functions as a single regulatory entity, in which Cbf11 acts as a DNA-binding subunit. Phylogenetic distribution of Cbf11 and Mga2 homologues showed that most Ascomycota species lack a Cbf11 homologue while Basidiomycota species possess both proteins, and S. pombe is an exceptional Ascomycota species containing both proteins. Cbf11 supposedly function exclusively together with Mga2 in organisms expressing both proteins, whereas Mga2 can operate independently of the Cbf11 in most Ascomycota species.

Although experiments are performed thoroughly and the presented results are largely convincing, the authors fail to provide direct evidence indicating that Cbf11 and Mga2 function as a single entity. While the findings strongly indicate that Cbf11 and Mga2 function in the same regulatory pathway, they do not necessarily indicate that they form a protein complex to function as a single entity. Unless additional evidence directly indicating physical interaction between Cbf11 and Mga2 is shown, the authors should tone down the claim and discuss other possibilities.

Major comments
Figure 4: Though the authors claim that sensitivities to TBZ and CPT in double deletion mutants are non-additive, the double deletion mutant cells appear to be more sensitive than mga2 single deletion mutant cells, especially on medium containing higher concentration of TBZ/CPT. This reviewer suggests the authors to perform more quantitative analysis to clarify this point. For example, the sensitivities may be quantitatively examined by measuring cell viability as a colony formation ability after exposure to TBZ/CPT for a certain period of time.

We have conducted the requested colony-forming unit assay to assess the viability of the WT, *Δcbf11*, *Δmga2*, and *Δmga2 Δcbf11* cells on plates containing CPT and TBZ (presented as Supplementary Figure S4). The results of the analysis indicated that the sensitivity of the double deletion mutant was not significantly enhanced in comparison to the *Δmga2* cells.

Page 7 line 24: While the authors state "these results are compatible with a scenario where Cbf11 and Mga2 form a complex that activates the expression of lipid metabolism genes", the results does not necessarily indicate that these proteins form a complex. It is equally possible that these proteins functions separately in a same regulatory pathway. For example, Cbf11 would modify chromatin structure of a target gene, and such modification would be required for DNA-binding of Mga2. Indeed, failure to detect physical interaction between these proteins, which is discussed in page 8-9, may suggest that Cbf11 and Mga2 function separately as distinct entities.

Thank you very much for pointing out this alternative scenario. Since we were indeed not able to prove the physical interaction between Cbf11 and Mga2, we now avoid the claim that Cbf11 and Mga2 function as a single entity in the revised version of manuscript. We have rephrased the problematic sentences to state that Cbf11 and Mga2 most likely work in the same regulatory

pathway. Also, we now mention in the Discussion multiple hypothetical mechanisms of joint functioning of Cbf11 and Mga2.

Minor comments

In Discussion (first paragraph), the authors claim that they have established Mga2 as a novel regulator of mitotic progression, but does not precisely explain which aspect of mitotic progression is regulated by Mga2. As various abnormalities, such as errors in DNA replication, chromosome segregation and spindle formation, result in the 'cut' phenotype, role of Mga2 in mitotic progression remain obscure. The authors should discuss the possible mitotic role of this protein in more detail.

We now discuss the presumed role of Mga2 in mitotic progression in the Discussion:

“It is tempting to speculate that Mga2 contributes to regulation of mitotic progression in the same way as Cbf11 does, since Mga2 mirrors all tested functions of Cbf11, as shown in this study. We have recently shown that proper cohesin dynamics and chromatin structure, i.e. factors that are altered in the *Δcbf11* mutant, contribute to mitotic fidelity (Vishwanatha et al. 2023).”

Reviewer #3 (Significance (Required)):

This study expands previous findings made by the authors' group and others, and clearly shows that Cbf11 and Mga2 function together in a same pathway to regulate transcription of genes in lipid metabolism although physical interaction between them remains unclear. Their novel findings may be of great interest to researchers in transcriptional regulation and lipid metabolism.
